# Chip-Based MEMS Platform for Thermogravimetric/Differential Thermal Analysis (TG/DTA) Joint Characterization of Materials

**DOI:** 10.3390/mi13030445

**Published:** 2022-03-16

**Authors:** Wenhan Zhou, Xinyu Li, Fanglan Yao, Haozhi Zhang, Ke Sun, Fang Chen, Pengcheng Xu, Xinxin Li

**Affiliations:** 1 State Key Laboratory of Transducer Technology, Shanghai Institute of Microsystem and Information Technology, Chinese Academy of Sciences, Shanghai 200050, China; zwh@mail.sim.ac.cn (W.Z.); xy_li@mail.sim.ac.cn (X.L.); yaofanglan@mail.sim.ac.cn (F.Y.); haozhi.zhang@mail.sim.ac.cn (H.Z.); sunke@mail.sim.ac.cn (K.S.); fchen01@163.com (F.C.); 2 School of Microelectronics, University of Chinese Academy of Sciences, Beijing 100049, China; 3 Shanghai QST Corporation Limited, Shanghai 200050, China

**Keywords:** thermogravimetric analysis, differential thermal analysis, resonant cantilever, thermopile, thermal decomposition

## Abstract

Combined use of thermal analysis techniques can realize complementarity of different characterization methods. Comprehensive thermal analysis with both thermogravimetric analysis and differential thermal analysis (TG/DTA) can measure not only mass change of a sample but also its temperature change during programmed heating-induced reaction or phase transition processes, thereby obtaining multiaspect thermal information of the material such as dehydration, structural decomposition, phase change and thermal stability. This study proposes and develops a MEMS chip-based TG/DTA microsystem that integrates both programmed heating and detecting elements into a TG chip and a DTA chip to enable the microinstrument performing TG/DTA joint characterization under microscope observation. The TG chip contains a self-heating resonant microcantilever to measure heating-induced mass change of a sample and the DTA chip is with a microheater and a temperature-detecting thermopile integrated on a suspended thermal-insulating diaphragm. Only nanogram and microgram-level samples are needed for the TG and DTA chips, thereby achieving safe measurement to energetic materials such as strong oxidants. The chip-based microinstrument surpasses the state-of-the-art commercial TG/DTA instruments that have, in the long term, suffered from large sample-amount (milligram level) requirements and have been unable to measure energetic materials. Compared with commercial instruments, the chip-based microinstrument is advantageous given its more accurate analysis, much higher heating rate, much smaller instrument volume and much lower power consumption, etc. The microinstrument has been fabricated by using wafer-level MEMS techniques. Testing results show that the mass-detection sensitivity of the TG-chip is as high as 0.45 Hz/pg in air and the temperature sensitivity of the DTA chip achieves 2.9 mV/K under the high heating rate of 25 °C/s. The strong oxidant of KMnO_4_ is analyzed with the TG/DTA joint characterization under microscopic observation. At the same time as microscope observation of the thermal decomposition phenomena, two-step thermal decomposition process of KMnO_4_ is identified and the thermal decomposition temperatures are obtained. The TG/DTA microinstrument is promising to be applied for study of various materials.

## 1. Introduction

Material thermal analysis technologies are frequently used to evaluate temperature-dependent properties of substances under programmed heating, which can be applied for the characterization and investigation of structure decomposition, thermal stability and phase transition, food safety assessment and drug evaluation [1,2,3,4], etc. By using the method of programmed temperature increase, various types of thermal analysis techniques can be used such as thermogravimetric analysis (TG or TGA) [5], differential thermal analysis (DTA) [6], differential scanning calorimetry (DSC) [7] and thermomechanical analysis (TMA, DMA) [8,9].

TG is a classic characterization technique for measuring the relationship between mass change of a sample and programmably heated temperature under a certain atmosphere. The technique is often used to study heating-induced dissociation, dehydration, decomposition and oxidation/reduction of substances by measuring the temperature related mass change. The disadvantage lies in that it is difficult to obtain the temperature-related heat-change process such as physical transition temperature of the sample (phase transition of metal, glass transition temperature of polymers, etc.) and the endothermic properties of a chemical reaction, where not only mass but also heat will change.

DTA is a technique for measuring the heat release or absorption-induced temperature change of a sample during the programmed temperature-increasing process under a certain atmosphere, which is often used to determine the special temperature of a sample during endothermic or exothermic phase transition or reaction. The disadvantage of DTA lies in that it is difficult to quantitatively obtain mass change during the test, such as chemical composition change and content (e.g., moisture) of the sample.

Therefore, joint characterization by using both TG and DTA has complementary advantages to obtain comprehensive thermal analysis information [10], thereby meeting the requirement of more accurate characterization to rapidly develop new functional materials [11].

It should be noted that the sample consumption of state-of-the-art commercial TG/DTA dual-function instruments is generally at the milligram level. The needed large amount of sample does cause difficulties in uniformly heating the sample during rapid heating, as well as inevitably leading to uneven temperature distribution and inaccurate measurement [12]. If the heating rate is slowed down, the time-consuming experiment will negatively affect the efficiency of TG/DTA. On the other hand, the required large sample amount also makes it too risky to analyze hazardous samples such as strong oxidants or explosives, where the analysis instrument may be damaged by severe exothermic reaction or even blast during heating. In addition, commercially available TG/DTA instrument uses closed furnace to heat the bulky sample, so it is difficult to put the sample under a microscope for in situ observation of the phase transition or structure evolution of the tested material. Finally, the core components of a commercially available TG/DTA instrument include a sample container for heating, a thermos balance for weighing during heating, temperature-measurement device and a cooling system, etc. Thus, the complex structures make the instrument quite expensive.

To solve the above-mentioned problem, herein a TG/DTA microsystem is proposed with all the core components fully integrated into a specifically designed micromachined resonance-cantilever chip for TGA (named MR-TGA) and a thermopile-embedded suspending diaphragm MEMS chip for DTA. The size of the TG/DTA microsystem based on the two MEMS chips is as small as centimeter level. The chip-based TG/DTA microsystem can be put under a microscope to observe the sample changes during the joint thermal analysis. Moreover, the samples can easily be loaded on the chips under the microscope by using a micromanipulator. The required sample amount is only at the level of 10s of nanograms (ng) and micrograms (μg) for MR-TGA and DTA, respectively. Ultrasensitive microchips loaded with a tiny amount of sample are advantageous in high-speed heating and high-efficiency accurate analysis. More importantly, the required tiny amount of sample makes the TG/DTA microsystem safe to use for thermal analysis of dangerous explosive substances such as strong oxidants. All these advances make the microsystem surpasses the market-available TG/DTA instrument.

## 2. Design of the Chip-Based TG/DTA Microsystem

**Figure 1:**
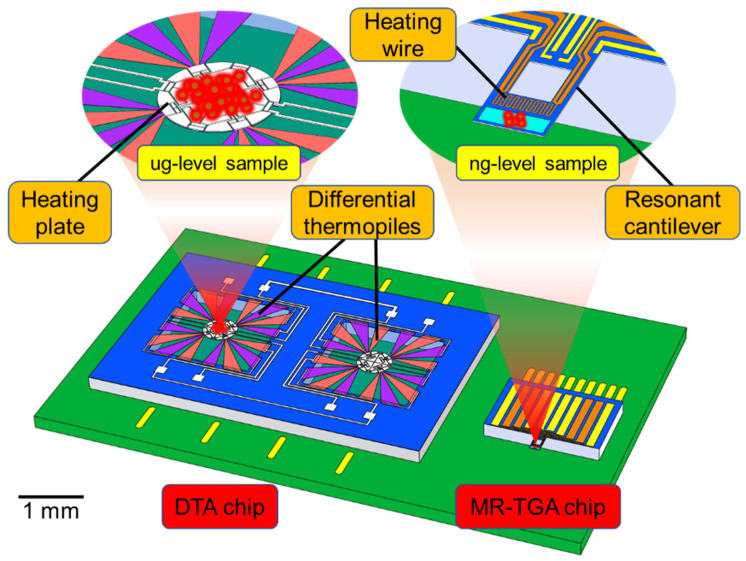
3D schematic of the proposed TG/DTA microsystem.

**Figure 2:**
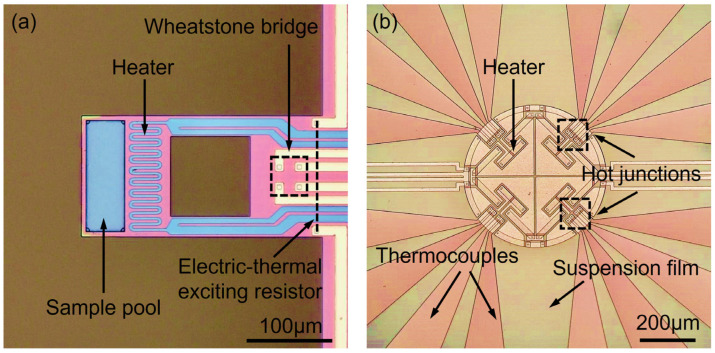
Top-view microphotograph showing the layout of the fabricated TG/DTA chips. (**a**) Cantilever beam of MR-TGA. (**b**) DTA thermocouple and heating plate in one suspended diaphragm.

Figure 1 shows the 3D schematic of the proposed TG/DTA microsystem, where the MR-TGA chip and the DTA chip are surface-mounted on a printed circuit board (PCB). In the DTA chip, two identical heating diaphragms integrated with identical thermopile layout are designed, with one thermopile-contained heating diaphragm for sample measurement and another one for temperature compensation. Figure 2 shows the top-view layout of the components integrated in the fabricated TGA chip and the DTA chip. As shown in Figure 2a, the dimensions of the silicon-resonant cantilever are 290 μm in length, 140 μm in width and 3 μm in thickness. It consists of a sample-loading microreservoir and a heating metal resistor at the cantilever free end, a piezoresistive Wheatstone bridge for frequency detection and a resistor at cantilever root for electric–thermal resonance excitation. The size of the MR-TGA chip is 1.7 mm × 1.85 mm. As shown in Figure 2b, one suspended square-diaphragm consists of a central heating-plate with a diameter of 500 μm, where both a resistive microheater and the hot-terminals of the serially connected thermocouples are integrated. The cold-terminals are outside the suspended insulating diaphragm (side length = 1.56 mm) located at the silicon frame heat-sink. The total size of the DTA chip is 3 mm × 5 mm.

Compared with the bulky commercial TG/DTA instrument, the proposed TG/DTA chip-based microsystem is only at centimeter scale. The smaller heating size helps to realize a much faster heating rate, where the TG/DTA microsystem can measure and control the temperature change within milliseconds. For maintaining a uniform thermal distribution of the sample, the maximum heating speed can reach 25 °C/s.

### 2.1. Operating Principles of MR-TGA Chip

The traditional TGA instrument measures changes in gravity of a sample through a precision balance, while the proposed MR-TGA continually measures the mass change of sample during programmed heating by detecting the shift of resonant frequency of the cantilever beam. In the MR-TGA, a metal heating resistor located at the free end of the cantilever is used to heat the sample pool. After precalibration between the temperature and the resistance change of the heater, the heating current inside the resistor wire can be controlled to achieve uniform heating to the sample. At the same time, the cantilever beam is excited into resonance by flowing DC-biased AC current into the silicon electric–thermal exciting resistor [13] integrated at the root of the cantilever, and the resonance is maintained by using a phase-locked loop (PLL) interface circuit. When the mass of the sample changes due to the programmed heating-induced sample reaction, such as dehydration or decomposition, the frequency shift is recorded in real-time by using the integrated piezoresistive Wheatstone bridge. The relationship between mass and resonant frequency can be expressed as [13]

$$\frac{m_{effT}}{m_{eff0}}=\frac{\frac{1}{f_{1T}^{2}}-\frac{1}{f_{0T}^{2}}}{\frac{1}{f_{10}^{2}}-\frac{1}{f_{00}^{2}}}\times \left(1+\beta T\right)\left(1+\alpha T\right)$$

where $m_{eff0}$ is effective mass of the cantilever at the room temperature and $m_{effT}$ is effective mass when the temperature is heated to T; $f_{00}$ and $f_{10}$ are resonant frequencies of the cantilever without and with the sample, respectively, at the room temperature; $f_{0T}$ and $f_{1T}$ are resonance frequencies of the cantilever beam without and with the sample at the heated temperature T. Here, *α* is Poisson’s ratio of the cantilever material and *β* is temperature drift coefficient of Young’s modulus. For the silicon cantilever, the value of *α* is 2.6 × 10^−6^/°C and the value of *β* is −6 × 10^−5^/°C.

As shown in Figure 2a, there is a hollowed rectangular window formed between the sample heating area and the frequency readout piezoresistors. With the design, the heat flow from the cantilever end to the silicon frame goes through the two sides of the cantilever, thereby maintaining the area for accommodating the piezoresistors near room temperature. Finite-element simulation by using COMSOL shows that the piezoresistive area is safely below 100 °C when the sample area is heated to 650 °C.

### 2.2. Operating Principles of DTA Chip

The thermopile in the DTA chip relies on the Seebeck effect to detect temperature change. The hot-junctions of the thermocouples connected in serial are located at the central heating plate of the suspended diaphragm, and the cold-junctions are connected to the heat sink outside the square diaphragm. When the temperature on the heating plate changes, the thermopile generates a potential U according to the temperature difference ∆T between the hot and cold junctions, which can be expressed as [14]

$$U=N\left(\alpha_{B}-\alpha_{A}\right)∆T$$

where N is the number of thermocouple pairs, $\alpha_{A}$ and $\alpha_{B}$ are Seebeck coefficients of the two thermocouple materials. Herein, *n*-doped polysilicon and *p*-doped polysilicon are chosen as the two thermocouple materials, since such design achieves quite large difference in Seebeck coefficient for high temperature-detection sensitivity. The DTA chip consists of two diaphragms that have identically designed two heating plates and two thermopiles. On one heating plate, the sample will be loaded. In contrast, the other plate will be kept empty or hold a reference sample that is insensitive to heating. Through a preset linear heating program, the two heating plates are heated at the same rate; therefore, there will be a differential temperature output between the two thermopiles when the tested sample experiences exothermic or endothermic reaction during the programmed heating. The differential signal $U_{out}$ can be expressed as

$$U_{out}=N\left(\alpha_{B}-\alpha_{A}\right)\cdot \left(T_{1}-T_{2}\right)$$

where $T_{1}$ and $T_{2}$ represent the temperatures on the two heating plates, respectively. DTA can detect the phase transition temperature point of the sample or the temperature range of the chemical reaction during heating by calculating the output waveform of the differential signal. Such DTA can judge whether the phase transition or chemical reaction is endothermic or exothermic by reading a positive or negative differential signal.

## 3. MEMS Fabrication

**Figure 3:**
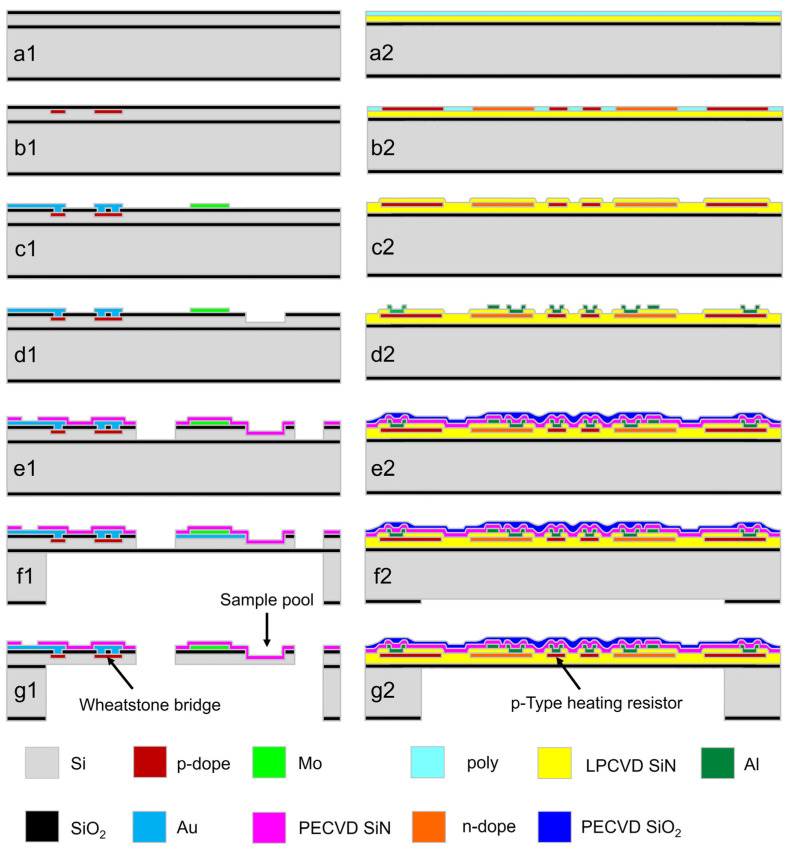
MEMS manufacturing process of the TG/DTA chips. The steps (**a1**–**g1**) are for the integrated resonant cantilever of MR-TGA chip and those from (**a2**–**g2**) are for the thermopile-integrated DTA chip.

Figure 3 details the MEMS fabrication steps for the two chips of the TG/DTA microsystem, where cross-sectional views are shown. The steps of Figure 3a1–g1 are for the resonant cantilever MR-TGA chip and the ones from Figure 3a2–g2 are for the thermopile DTA chip.

Figure 3a1: thermal oxidation is processed to (100) silicon-on-insulator (SOI) wafers, where the *n*-type active layer is 3 μm in thickness. Figure 3b1: piezoresistive Wheatstone-bridge and electro–thermal resistor are simultaneously formed by using *p*-type silicon doping technique for frequency-signal readout and resonance excitation, respectively. Figure 3c1: 10 nm/200 nm Cr/Au composite layer is developed by using sputtering and patterned for interconnection. Then, 200 nm-thick Mo thin-film is sputtered and patterned into the heating resistor. Figure 3d1: the sample loading pool is formed by a shallow etch with reactive-ion-etch (RIE). Figure 3e1: 300 nm SiN is formed by using plasma enhanced chemical vapor deposition (PECVD) as passivation layer. After patterning, cantilever shape is patterned and formed with RIE. Figure 3f1: backside silicon etch using deep RIE is performed to remove the substrate silicon beneath the cantilever. Figure 3g1: structural release of the cantilever is processed by etching off the buried SiO_2_ layer beneath the cantilever with buffered aqueous HF.

Figure 3a2: thermal oxidation is processed to the same (100) silicon wafer, which is then followed by low-pressure CVD (LPCVD) deposition of 300 nm low-stress SiN and 500 nm polysilicon. Figure 3b2: Boron and phosphorous ion implantation processes are sequentially implemented to form the *n*-type and *p*-type polysilicon microregions of the *n*/*p* polysilicon thermocouple and the *p*-type heating resistor. The resistance of *n*/*p*-poly is about 67 Ω/Square. Figure 3c2: the thermocouples and the heating resistors are patterned, and then deposition of 300 nm LPCVD SiN is followed. Figure 3d2: sputtering and patterning of 600 nm-thick Al interconnection is processed. Figure 3e2: sequential deposition of 300 nm SiN and SiO_2_ is processed by using PECVD. Figure 3f2 backside silicon etching window is patterned. Figure 3g2: deep RIE is used from backside to release the suspended diaphragm structure.

**Figure 4:**
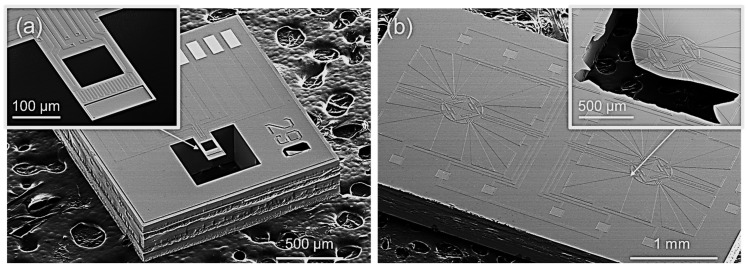
SEM images of the two chips for TG/DTA microsystem. (**a**) MR-TGA chip with the inset showing the magnified view of the cantilever. (**b**) Thermopile-integrated DTA chip with the inset showing the intentionally disrupted suspended diaphragm.

The fabrication process is at silicon wafer level for low-cost volume applications. Thanks to the tiny sample amount for heating and satisfactory thermal conducting properties of the silicon chips, complex heating and cooling parts are not required. Figure 4 shows the scanning electron microscope (SEM) images of the fabricated MR-TGA chip and thermopile-integrated DTA chip.

## 4. Calibration of the TG/DTA Microsystem

**Figure 5:**
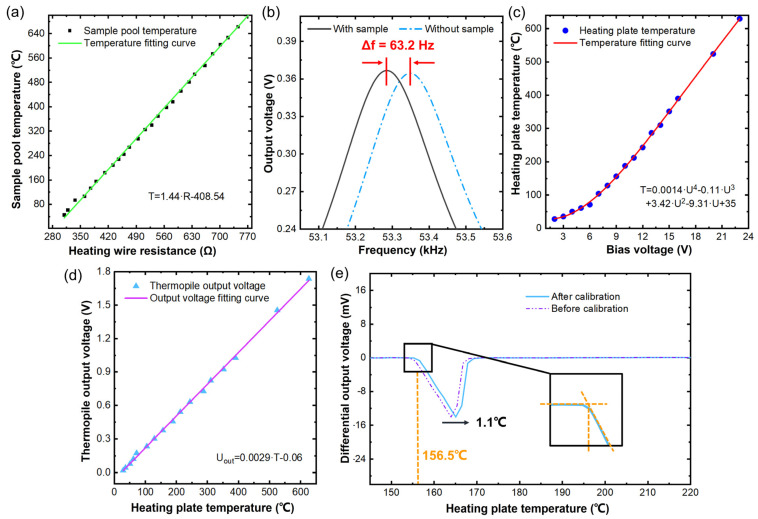
Temperature calibration and sensitivity test of the TG/DTA microsystem. (**a**) Calibrated MR-TGA resistance of the microheater versus temperature. (**b**) Mass sensitivity obtained by testing the frequency shift of MR-TGA with 140 μg sample. (**c**) Calibrated heating-plate temperature versus input voltage of heating resistor for DTA. (**d**) Thermopile output voltage versus heating-plate temperature in DTA. (**e**) Tested melting curve of indium for calibration of DTA.

The TG/DTA microsystem requires a temperature calibration process based on input programming. The heating regions of the two chips are noncontact measured by using an infrared camera with a spatial resolution set to 20 μm. For the MR-TGA chip, the relationship between the resistance value of the heater and the temperature of the sample pool is calibrated by using the infrared camera, with the results shown in Figure 5a. The obtained temperature coefficient of the microheater resistance (TCR) is calculated and applied as the control signal of the programmed heating process. Figure 5b shows the calibrated mass-detecting sensitivity of the MR-TGA. Tested in the air with 140 pg standard mass sample (polystyrene microspheres), the frequency shift of 63.2 ± 0.1 Hz indicates that the sensitivity at room temperature is 0.45 Hz/pg. The temperature-dependent mass sensitivity of the cantilever is tested, resulting in a negligible heating-induced sensitivity drift of 25 ppm/°C that mainly comes from the temperature coefficient of silicon Young’s modulus.

In the DTA chip, doped polysilicon is used as heating resistor, and the linearity of its TCR is not as good as that of metal resistor. By directly measuring the relationship between the input voltage of the heating resistor and temperature rise of the heating plate that is shown in Figure 5c, the fit voltage–temperature curve can be used to control the programmed heating process. Then, the sensitivity of the thermopile for temperature measurement is also obtained by detecting the temperature-dependent output signal of the thermopile. As shown in Figure 5d, the temperature sensitivity of the thermopile is 2.9 mV/°C ± 0.1% and the output voltage is linear with the temperature at the heating plate. The noise equivalent power (NEP) of the thermopile is calculated as 0.09 μW @ 400 Hz according to the thermal noise voltage and sensitivity [15].

The melting point of the standard calibration sample of indium is tested to confirm the temperature-detecting accuracy of the infrared thermal imager. Figure 5e shows the DTA curve of the indium during the heating-induced melting process. The thermopile readout melting point of indium is 155.4 °C, which is 1.1 °C lower than the known melting point of indium. Temperature calibration of the DTA chip is completed by using the results to adjust the programmed heating function. This experiment also shows that the temperature measurement accuracy of the infrared camera is sufficient for experiment.

Table 1 shows the performance comparison of our MR-TGA and DTA in this paper with reported micro-TGA and micro-DTA. It indicates that our chips have sufficient sample area, wide heating range and good sensitivity. Although device in [16] shows better sensitivity than other micro-TGA, it requires extra laser Doppler vibrometer which greatly increases the complexity of the test system.

## 5. TG/DTA Joint Characterization Results

**Figure 6:**
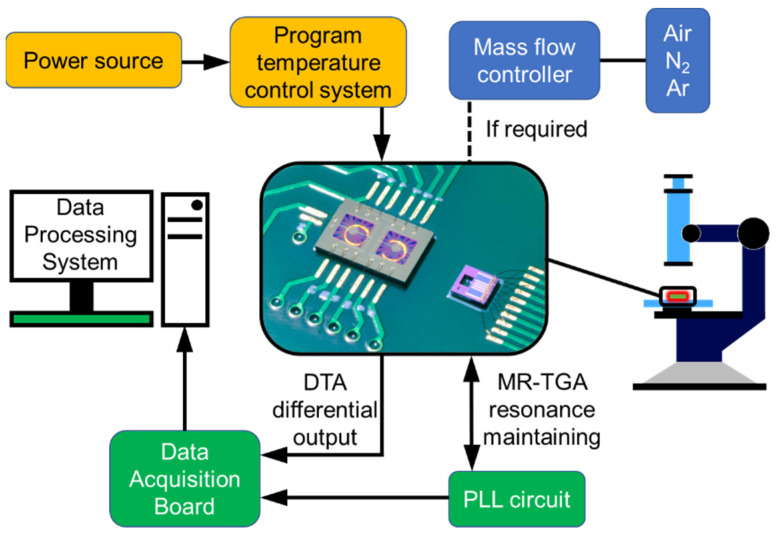
Schematic setup of the TG/DTA joint-characterization microsystem.

Figure 6 schematically shows the setup of the TG/DTA microsystem. To realize the program-controlled temperature rise, voltage-programming function of an Agilent E3631A power supply is employed by using the software control program. The voltage accuracy of the programmed power supply is 1 mV ± 0.1%, and the voltage switching rate is about 60 ms ± 1%. When the microsystem is heated linearly, the fastest heating rate can be 25 °C/s. Since the MEMS chip-based test system is quite small in size, it can be placed on the sample stage of an optical microscope to in situ observe the sample phenomenon during the heating induced reaction through the microscope lens. If necessary, the test system can also be placed into a gas chamber for analysis under different gas atmospheres. Signal is recorded with a NI USB-6366 data acquisition card. At the same time, a Lab-VIEW platform is used to complete the preprocessing of the differential output signal of DTA and the resonant frequency signal of MR-TGA. The sample is firstly dispersed in some solvent, such as water, to form a suspension. Then, the suspension can be loaded precisely into the sample pool using the inkjet printer under an optical microscope [19]. All MEMS chips are replaced after each test to avoid contamination by residual sample.

**Figure 7:**
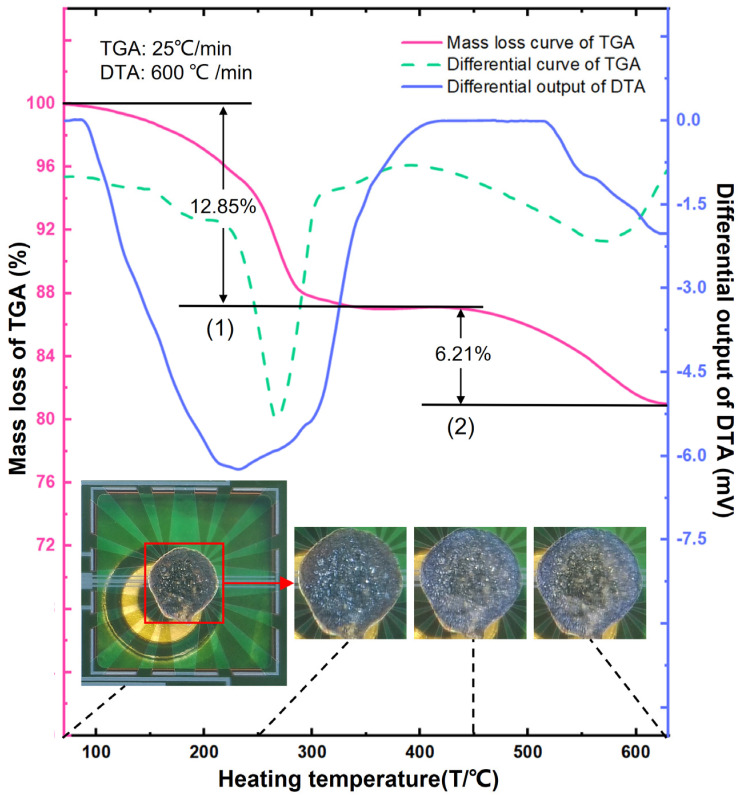
TG/DTA joint characterization results of KMnO_4_ by using the chip-based microsystem in air atmosphere. The microsystem is put under a microscope for simultaneous observation of morphologic evolution during the heating-induced two-step decomposition process, with the images of the sample in the thermopile chip inset in the figure.

Explosive, corrosive, or strongly oxidizing samples are difficult to test with market-available TG/DTA instruments due to the risk of contamination or even blast damage of the instrument by heating such samples at the mg level. In contrast, the herein-proposed TG/DTA microsystem requires only ng-level samples for TGA and µg-level samples for DTA, which are too small to pose severe explosion or other dangers. As shown in Figure 7, the TG/DTA microsystem is used to perform safe measurement to potassium permanganate (KMnO_4_), which is a typical hazardous chemical due to its strong oxidizing properties and explosiveness.

Figure 7 clearly shows the two mass-loss steps in the TGA curve of KMnO_4_, which indicates the continual heating induced two-step decomposition process of KMnO_4_ with the number marked in the figure. The differential results of the mass-loss curve (see the dotted green curve) also indicates the two steps. The corresponding decompositions are known as [20,21]

(1) $$10{\;\mathrm{KMnO}}_{4}\overset{\Delta }{\to }2.65{\;K}_{2}{\mathrm{MnO}}_{4}+\left[2.35{\;K}_{2}O\cdot 7.35{\;\mathrm{MnO}}_{2}\right]+6{\;O}_{2}↑$$

(2) $$2.65{\;K}_{2}{\mathrm{MnO}}_{4}+7.35{\;\mathrm{MnO}}_{2}\overset{\Delta }{\to }2.65{\;K}_{2}O+5{\;\mathrm{Mn}}_{2}O_{3}+3.825{\;O}_{2}↑$$

In step (1), the overall reaction includes the decomposition of ${\mathrm{KMnO}}_{4}$ and $K_{3}{\left({\mathrm{MnO}}_{4}\right)}_{2}$. According to the relative molecular mass change in the reaction Equation (1), the relative mass loss is calculated as 12.15% due to the generated oxygen gas, which agrees well with the measured mass loss of 12.85% in the TGA curve. In the step (2), the overall reaction includes sequential decomposition of $K_{2}{\mathrm{MnO}}_{4}$ and ${\mathrm{MnO}}_{2}$. The calculated mass loss based on the reaction Equation (2) is 7.59% and that tested value is 6.21%. Limited by the highest temperature tolerance of the Al metal wire, the heating program of the test system has to be stopped at 630 °C. Hence, the reaction in step (2) is not thoroughly completed, which may be why the tested mass loss was a little bit smaller than the calculated one.

The DTA curve confirms the TGA results, which distinguishes that the first decomposition occurs between 100 and 400 °C and the second decomposition occurs between 450 and 630 °C. More importantly, the DTA results clearly judge that the two reaction stages are endothermic by the negative sign of the output waveform. Through the combined test of both MR-TGA and thermopile DTA, the MEMS chip-based TG/DTA microsystem can real-time obtain the characterization results, such as the reaction temperature range, the composition changes and the judgment of KMnO_4_ thermal decomposition being an endothermic process.

Thanks to the small testing system for microscopic observation, the sample morphologic evolution during the two-step decomposition are real-time recorded which can be seen in the insets of Figure 7.

## 6. Conclusions

The proposed MEMS chip-based thermogravimetric/differential-thermal analysis (TG/DTA) joint-detection microsystem has been developed by using MEMS integration techniques. Compared with commercially available TG/DTA equipment, the microinstrument has advantages in its smaller size, lower manufacturing cost, faster heating rate, etc., thereby enabling rapid heating testing, real-time microscopic observation of the morphologic evolution of the material during programmed heating. Thanks to the required sample amount of nanogram and microgram levels for TGA and DTA, the TG/DTA microinstrument can analyze energetic materials that have historically been difficult to measure in commercially available TG/DTA instruments. The proposed TG/DTA microinstrument is expected to be widely used for the thermal analysis of various advanced materials.

## Figures and Tables

**Table 1:** Performance comparison between micro-TGA and micro-DTA.

	Reference	Working Principle	Sample Area (μm^2^)	Mass Level	Sensitivity	Temperature Drift or NEP	Maximum Temperature
TGA	Our work	Resonant cantilever	60 × 130	ng	0.45 Hz/pg	25 ppm/°C	900 °C
	[17]	Resonant cantilever	≈70 × 140	ng	0.16 Hz/pg	16 ppm/°C	647 °C
	[16]	Resonant cantilever with laser Doppler vibrometer	<9 × 53	pg	522 Hz/pg	35 ppm/°C	-
DTA	Our work	Thermopile on membrane	250^2^π	ug	2.9 mV/K	0.09 μW @ 400 Hz	630 °C
	[18]	Thermopile on cantilever	≈40×270	ug	0.03 mV/K	33 μW @ 5 kHz	400 °C
	[15]	Thermopile on membrane	250^2^π	ug	4 mV/K	0.1 μW @ 400 Hz	450 °C
